# Investigating respiratory infection as a post-COVID-19 condition using a large passive surveillance cohort from Track PCC

**DOI:** 10.1186/s12879-026-13994-0

**Published:** 2026-07-16

**Authors:** Marina Oktapodas Feiler, Recai M. Yucel, Bari J. Dzomba, Resa M. Jones

**Affiliations:** 1 Department of Epidemiology and Biostatistics, Barnett College of Public Health, Temple University, 1210 West Berks Street, Paley Hall, Philadelphia, PA 19122-6088 USA; 2 Department of Epidemiology and Environmental Health, School of Public Health and Health Professions, University at Buffalo, Buffalo, NY USA; 3 Department of Health Services Administration and Policy, Bartnett College of Public Health, Temple University, Philadelphia, PA USA; 4 Fox Chase Cancer Center, Temple Health, Temple University, Philadelphia, PA USA

**Keywords:** SARS-CoV-2, Long COVID, Post-COVID conditions, Medical record, EHR

## Abstract

**Background:**

This study examined risk factors for post-COVID respiratory infection using data from the *Tracking the Burden*,* Distribution*,* Impact of Tracking Post-COVID-19 Conditions in Diverse Populations for Children*,* Adolescents*,* Adults* (Track PCC) passive surveillance cohort.

**Methods:**

This retrospective study included adult Temple Health patients in Philadelphia, Pennsylvania with SARS-CoV-2 (COVID-19) infections from March 2020 to December 2022 and ≥ 90 days follow-up. COVID-19 infection was identified via laboratory testing, billing codes, or clinical documentation. The primary outcome was post-COVID respiratory infection identified by billing codes. Predictors included social, clinical, and COVID-related correlates. Adjusted logistic regression models were used on 17,539 complete cases and multiple imputed datasets (*n* = 45,513) with SuperMICE.

**Results:**

In complete-case analysis, uninsured coverage, Alpha variant, total comorbidities, and hospitalization were associated with lower odds of respiratory post-COVID conditions (ORs: 0.02–0.99). Dual Medicare/Medicaid and current smokers, increased odds (ORs: 1.33–1.58). Imputed analyses showed consistent results with and additionally observed higher odds of respiratory infection among those with Medicaid and higher number of vaccinations, and lower odds among those of any non-white race, Hispanic ethnicity.

**Conclusions:**

Temple Track PCC findings identify high-risk populations and underscore the utility of advanced imputation in surveillance-based research.

**Supplementary Information:**

The online version contains supplementary material available at https://doi.org/10.1186/s12879-026-13994-0.

## Introduction

Post-COVID conditions (PCC), also known as long COVID, are defined as the continuation or development of new symptoms at least three months after the initial SARS-CoV-2 (COVID-19) infection, lasting for at least two months and not attributable to alternative diagnoses [1]. These conditions can affect multiple organ systems, including respiratory, cardiovascular, neurological, metabolic, and hematological, and are associated with functional impairments, reduced quality of life, and increased healthcare utilization [2, 3]. Given their broad impact, PCC represents a growing public health challenge that places substantial burden on healthcare systems [4–6].

Respiratory infections remain one of the most important contributors to morbidity and mortality in adults worldwide and in the United States [7–9]. Increasing evidence suggests that individuals with prior COVID-19 infection may be at heightened risk of developing subsequent respiratory infections, even after recovery from mild or moderate illness [10–12]. Proposed mechanisms to explain this increased vulnerability include persistent viral reservoirs, immune dysregulation, heightened systemic inflammation, alterations in host-defense responses, and changes in bacterial virulence [13–15]. Immunological and histopathologic studies suggest lasting lung damage, including fibrosis [16, 17]. Collectively, these processes may predispose individuals to subsequent viral and bacterial respiratory infections. Epidemiological studies support this concern, observing a higher risk of subsequent and new respiratory infections among those with previous COVID-19 infections compared with those never infected [10], and correlations between COVID-19 severity and new respiratory infections among COVID-19 survivors [18]. These findings underscore the importance of recognizing respiratory infection as a distinct and clinically meaningful PCC outcome, particularly in vulnerable groups such as older adults and individuals with comorbidities [19, 20].

Despite these concerns, research on post-COVID respiratory infections remains limited, and predictors of risk remain poorly defined. Understanding determinants of post-COVID respiratory infections and complications is essential for patient care and for guiding population-level prevention strategies. The present study was uniquely positioned to investigate these determinants due to the large, diverse, and longitudinal sample of patients in North Philadelphia. This work also employs rigorous methods to leverage complex electronic health record (EHR) data and improve analytic validity [21, 22]. The objective of this study is to evaluate the association between demographic, social, clinical, and COVID-related correlates and post-COVID respiratory infection in a large and diverse population of adults in North Philadelphia.

## Methods

The parent study, *Tracking the Burden*,* Distribution*,* Impact of Tracking Post-COVID-19 Conditions in Diverse Populations for Children*,* Adolescents*,* Adults* (Track PCC), is funded by the Centers for Disease Control and Prevention (CDC). It is a multi-site surveillance initiative designed to identify and monitor long-term health effects following COVID-19 infection [23]. Track PCC employs both active and passive surveillance to capture data on individuals with self-reported or documented COVID-19 infection and assess the incidence and patterns of PCCs across diverse U.S. populations. This study has been approved by Institutional Review Board at Temple University, Temple IRB# 30,911, consent to participate declarations were not applicable.

The present analysis utilized passive surveillance data from Temple Health patients between March 1, 2020 and December 31, 2022 for adults who were identified through laboratory-confirmed COVID-19 infections (polymerase-chain reaction, PCR, or antigen tests), International Classification of Diseases (ICD) codes, clinical diagnoses, and/or self-reported documentation in EHR. Records from the Philadelphia Department of Public Health were used to capture any additional COVID-19 diagnoses missed in the EHR. Eligible participants needed to have at least one healthcare encounter at Temple Health in the year prior to COVID-19 infection, with follow-up beginning 30 days post-infection and continuing for up to 18 months. As of October 31, 2024, the data included 108,596 unique COVID-19 cases, after excluding those with no clinical encounter data the overall sample included *N* = 45,513.

Surveillance data, including clinical encounters, diagnoses, laboratory reports, and vaccination records, were collected from the EHR and public health sources, then securely transmitted to a centralized database for quality control and harmonization.

Six primary datasets were used: (1) a demographic dataset capturing age, sex, body mass index (BMI), race, and ethnicity; (2) a vaccination dataset detailing vaccine status, doses, type, and dates; (3) a diagnosis dataset containing ICD codes and categories used to identify post-COVID conditions (PCCs) and comorbidities; (4) a geographic dataset linking residential location to the CDC Social Vulnerability Index (SVI) and population density at the census tract level; (5) a medication dataset documenting prescribed/administered medications with dates; and (6) a hospitalization dataset describing clinical care setting (ambulatory, non-critical hospitalization, or critical care), as a proxy for COVID-19 severity.

Supplemental Figure 1 presents the Directed Acyclic Graph (DAG) that guided the analytical approach by visually outlining the hypothesized relationships between key variables, in the context of respiratory infections following COVID-19 infection.

### Outcome variable

Respiratory infection diagnosis (RID) PCC was identified using a dual approach. First, respiratory infection–related ICD-10 codes (e.g., J189, J209, J40, J22, J80, J9600, J8410; see Supplementary Table 1) were used. If no qualifying ICD-10 diagnosis was recorded, prescriptions for medications commonly used to treat respiratory infections, such as inhaled steroids, were used as indicators, guided by a Temple University co-investigator and infectious disease physician.

Only the first respiratory infection–related event occurring after COVID-19 infection was considered, defined as the earliest diagnosis date or the earliest prescription date for a relevant respiratory medication. Prescribed or administered medication records were used to capture medication type and date. Respiratory medications included inhaled steroids and other inhaled therapies.

A binary outcome variable was constructed classifying those with a RID ICD-10 code or medication use occurring before the initial COVID-19 infection coded as 0 as this was indicative of a prior RID before COVID-19. Then those with an RID ICD-10 code or medication use were coded as 1, indicating a PCC RID. Lastly, individuals without evidence of RID or medication use were classified as missing. Due to limitations of EHR data capture, this resulted in a substantial proportion of missing outcome data.

### Potential correlates of post-COVID respiratory infection

Measures of demographic, social, clinical, and COVID-related correlates were included based on factors previously identified as relevant to COVID-19 disparities and health outcomes and by using our DAG (Supplementary Figure 1) [29, 31].

### Demographic correlates

The Track PCC parent study constructed a combined race/ethnicity variable. Individuals were first categorized based on Hispanic ethnicity, and then by race. Those identified as Hispanic were grouped as “Any race, Hispanic” regardless of their reported race. Non-Hispanic individuals were classified into one of the following racial categories: White, Black/African American, Asian/Asian American, American Indian/Alaska Native, Native Hawaiian/Pacific Islander, Other or Multiracial, or Unknown race. Individuals with missing information for both race and ethnicity were grouped as “unknown race and unknown ethnicity.” However, for this study, due to small sample sizes for several race/ethnicity categories in the Temple Track PCC data, we collapsed Asian, American Indian, Native Hawaiian, Other race, and Unknown race into a single category labeled “Other”. Biologic sex was characterized as male and female, pulled from the EHR. The age variable was developed using date of birth and date of post-COVID respiratory infection diagnosis in the EHR.

Insurance status included private, Medicare, Medicaid, CHIPS, or other non-military public, dual Medicare and Medicaid, Tricare, and other uninsured categories. Due to sample size, for individuals with Tricare coverage, we re-categorized the variable based on age: those aged 65 or older were grouped with the Medicare category, while those under 65 were grouped with Medicaid.

### Social correlates

Smoking status was a self-reported measure of the individual’s smoking behavior originally categorized as current every day, current some days, former, never smoker or smoker but current frequency unknown. Individuals who reported smoking every day, some days, or with unknown frequency were combined into a single category of “current smoker.” This yielded a three-level categorical variable: current smoker, former smoker, and never smoker.

SVI was created utilizing area-level characteristics covering four themes, including socioeconomic status, household characteristics, racial and ethnic minority status, and housing type and transportation [24]. Patient addresses were geocoded and linked with the US Census to define these themes and develop the SVI variable, this variable has a range of 0 to 1 with 0 indicating low vulnerability and 1 indicating high vulnerability [24].

### Clinical correlates

BMI was calculated using weight and height in EHR closest to RID diagnosis. To quantify the number of comorbidities, we computed the total number of distinct Charlson comorbidity categories present for each patient using ICD-10 diagnosis codes. This count-based approach, derived from the Charlson Comorbidity Index (CCI), reflects the overall presence of chronic disease without applying comorbidity weights [32]. Although CCI does not have a fixed range, a score of 0 indicates no comorbidities were found, 1–2 are classified as mild, 2–4 are classified as moderate, and ≥ 5 is considered severe [25].

### COVID-related correlates

Information about the date of COVID-19 infection (given ICD-10 diagnosis date or self-report date), SARS-CoV-2 variants, COVID-19 severity, prescribed respiratory medication received and first date of medication, total number of COVID-19 vaccine doses, number of comorbidities was collected.

SARS-CoV-2 variants were defined using the CDC classifications based on dates of diagnosed or self-reported infection [26]. Those diagnosed/self-reported between 1/2020 and 3/2021 were coded as having the “original” variant, between 3/2021 and 6/2021 were coded as “Alpha” variant, between 6/2021 and 12/2021 were coded as “Delta” variant, and between 1/2022 and 10/2024 were coded as “Omicron.”

COVID-19 severity was defined using hospital admission type. This was a categorical variable with three levels; ambulatory only (lowest severity), hospitalization non-critical (moderate severity), and hospitalization critical (highest severity). The number of COVID-19 vaccine doses was captured in EHR and vaccine registries (range: 0–4 doses).

### Statistical analysis

Descriptive analyses examined the distributions and frequencies of our variables and identified quantity of missingness in our dataset. Mean, standard deviation (SD), and interquartile range (IQR) were calculated for our continuous variables. Correlations among COVID-19 variant, disease severity, age, comorbidity burden, and vaccination uptake/dosess were evaluated using a Pearson correlation heat map to assess multicollinearity. Overall, correlations among correlates were low to moderate (*r* = 0.00–0.50), and the correlation for COVID-19 variant and disease severity was weak (Cramer’s V = 0.06), suggesting multicollinearity was unlikely to affect our model estimates. For this reason, all variables were retained based on their distinct clinical and epidemiologic relevance to our overall objective.

### Complete-case multiple logistic regression

To assess measures of association, we first fit a logistic regression model using only complete-case data with respect to all variables in the substantive model for respiratory infection. Of the 45,513 observations, only 17,539 (37%) cases had complete data and were used to fit a logistic regression model for estimating the odds of RID. Our assessment of missingness, using a test developed by Little [20], showed missing completely at random is an invalid assumption in our data [21]. This indicates that complete-case analysis would likely produce biased and inefficient estimates. Parameter estimates, standard errors, and p-values for each predictor in the complete-case analysis are provided.

### Supervised learning for chained imputation (SuperMICE) multiple logistic regression

Supervised Multiple Imputation by Chained Equations (SuperMICE) integrates ensemble machine learning into the traditional Multiple Imputation by Chained Equations (MICE) procedure. Essentially, SuperMICE [27] utilizes variable-by-variable imputation method as implemented in MICE [28] with machine learning routines to improve accuracy, flexibility, and robustness of analytic validity [40, 41]. Similar to MICE, a major attraction of SuperMICE pertains to its variable-by-variable imputation: each incomplete variable is iteratively imputed using an appropriate parametric regression model (e.g., linear regression for continuous variables, logistic regression for binary variables) or nonparametric approaches such as predictive mean matching or random forests. To address limitations of standard MICE, including model misspecification or computational failure due to high dimensionality, SuperMICE replaces the default imputation regression model for each incomplete variable with a Super Learner ensemble machine learning framework as implemented in the R package *mice* [28, 29]. At each iteration, candidate learners are fit to the observed data and combined using cross-validated non-negative least-squares weights to minimize prediction error, yielding an optimally weighted prediction model for imputation. Poorly performing learners receive zero weight and are effectively excluded. The resulting ensemble generates predicted values for missing observations. For example, the *glmnet* learner contributes regularized regression predictions with tuning parameters selected through 10-fold cross-validation. The ultimate goal of this is to minimize the overall cross-validated prediction error,^38^ and the resulting prediction model is used in place of standard regression models within the chained equations process, producing imputations that more accurately reflect the underlying data distribution.

Because SuperLearner is a prediction-based rather than posterior-sampling approach, kernel smoothing (Gaussian kernel with jackknife bandwidth selection) is used to estimate local prediction variance. Multiple imputations are then generated by sampling from a normal distribution centered on each predicted value, with variance reflecting imputation uncertainty. This process is repeated to create multiple completed datasets (e.g., 10 imputations).

The analysis model (logistic regression for PCC respiratory infection diagnosis) was fit separately within each imputed dataset. Estimates and standard errors were then combined using Rubin’s rules [30, 31], whereby regression coefficients are averaged across imputations and variance estimates incorporate both within-imputation and between-imputation variability. This approach accounts for uncertainty arising from both sampling and missing data.

Several diagnostics were used to assess the validity and stability of the imputation procedure. Trace plots of the means and standard deviations of imputed values were examined to confirm adequate mixing across iterations without systematic trends. We also verified that imputed distributions closely resembled observed-data distributions, consistent with the missing-at-random assumption. Had substantial discrepancies been observed, the SuperLearner library would have been expanded to include additional learners; however, this was not necessary.

Finally, we evaluated imputation performance using the fraction of missing information (FMI) [32], which quantifies the proportion of uncertainty in the final inference attributable to missing data. FMI provides insight into the information lost due to missingness, the extent to which imputation reduces that uncertainty, and the impact of missing data on statistical inference, including considerations regarding the number of imputations and the amount of information recovered from incomplete observations. Estimated FMI for the regression estimates reported Table 3 ranged between 0.00014 and 0.01427, indicating that the sampling of the missing data (i.e., multiple imputations) had relatively very small impact on the regression coefficients.

### Interaction

To evaluate potential effect modification, interaction analyses were conducted by including interaction terms in the multivariable logistic regression models. Specifically, interaction terms for age and number of COVID-19 vaccine doses and total comorbidities and COVID-19 severity were assessed in both the complete-case and SuperMICE analyses to evaluate the consistency of findings across analytic approaches.

## Results

Table 1 provides the descriptive statistics of the study variables stratified by those with a PCC RID, those without a PCC RID, and those missing a PCC RID among a total of 45,513 individuals. Median (IQR) age for those with and without a PCC RID were similar at 56 years (42, 67) and 57 years (43, 69), respectively, and was lower for those missing PCC RID at 46 years (32, 62). More females (62%), current smokers (23%), and individuals with insurance (91.5%) were diagnosed with a PCC RID. BMI and SVI were similar across all groups, indicating median BMI as high overweight/obese and high social vulnerability. The median number of COVID-19 vaccine doses was similar across groups at 2, IQR: 2, 3.

**Table 1:** Summary statistics for study variables: Temple Track PCC, Philadelphia, Pennsylvania, March 2020 – December 2022 (*N* = 45,513)

Variables	PCC RID *N* = 16,284 *n* (%)	No PCC RID *N* = 3,706 *n* (%)	Missing PCC RID *N* = 25,523 *n* (%)	*p*-value^a^
Sex				
Female Male Missing	10,112 (62.1) 6172 (37.9) 0 (0)	1914 (51.6) 1792 (48.4) 0 (0)	14,893 (58.4) 10,629 (41.6) 1 (< 0.1)	< 0.001
Race/Ethnicity				
White, non-Hispanic Black/African American, non-Hispanic Other, non-Hispanic Any race, Hispanic Missing	3362 (20.6) 6738 (41.4) 1055 (6.5) 4944 (30.4) 185 (1.1)	645 (17.4) 1523 (41.1) 342 (9.2) 1073 (29.0) 123 (3.3)	5740 (22.5) 10,336 (40.4) 2127 (8.3) 6373 (25.0) 947 (3.7)	< 0.001
Insurance Status				
Private Insurance Medicare Medicaid, CHIPS, or other non-military public Dual Medicare/Medicaid Other^b^ Uninsured Missing	1995 (12.3) 2659 (16.3) 6000 (35.7) 3881 (23.8) 365 (2.2) 1380 (8.5) 4 (< 0.1)	316 (8.5) 466 (12.6) 838 (22.6) 407 (11.0) 109 (2.9) 1569 (42.3) 1 (< 0.1)	4371 (17.1) 2872 (11.3) 8032 (31.5) 2095 (8.2) 688 (2.7) 7446 (29.2) 19 (< 0.1)	< 0.001
Smoking Status^c^				
Never Former Current Missing	7309 (44.9) 4831 (29.6) 3754 (23.1) 390 (2.4)	2024 (54.6) 932 (25.1) 618 (16.7) 132 (3.6)	14,159 (55.5) 4753 (18.6) 5087 (19.9) 1524 (6.0)	< 0.001
Age (years; median, IQR)	56 (42, 67)	57 (43, 69)	46 (32, 62)	< 0.001
Missing	0 (0)	0 (0)	0 (0)	
Social Vulnerability Index (median, IQR)^d^	9 (8, 10)	9 (8, 10)	9 (8, 10)	< 0.001
Missing	424 (2.6)	170 (4.6)	837 (3.3)	
Body Mass Index (median, IQR)	30 (26, 36)	30 (25, 36)	29 (25, 34)	< 0.001
Missing	52 (0.3)	20 (0.5)	1070 (4.2)	
COVID-19 Variant^e^				
Original Alpha Delta Omicron Missing	6977 (42.8) 1550 (9.5) 2257 (13.9) 5500 (33.8) 0 (0)	1630 (44.0) 541 (14.6) 580 (15.7) 955 (25.8) 0 (0)	8900 (34.9) 2123 (8.3) 4326 (16.9) 10,174 (39.9) 0 (0)	< 0.001
Total Comorbidities (median, IQR)^f^	2 (1, 4)	1 (1, 3)	0 (0, 1)	< 0.001
Missing	0 (0)	0 (0)	0 (0)	
Number of COVID-19 Vaccinations (median, IQR) Missing	2 (2, 3) 4713 (28.9)	2 (2, 3) 1895 (51.1)	2 (2, 3) 11,652 (45.7)	< 0.001
Severity Status due to COVID-19^g^				
Ambulatory Hospitalized, not critical Hospitalized, critical Missing	12,058 (74.0) 3237 (19.9) 989 (6.1) 0 (0)	782 (21.1) 2115 (57.1) 809 (21.8) 0 (0)	22,628 (88.7) 2103 (8.2) 792 (3.1) 0 (0)	< 0.001

^a^Kruskal-Wallis rank sum test; NA; Pearson’s Chi-squared test

^b^Other insurance category in Track PCC EHR data includes Temple-specific categories such as “Temple Athletic”, “Temple Med Student”, or “worker’s comp”

^c^Self-reported smoking behavior categorized as never smoker, former smoker, and current smoker

^d^Based on the CDC Social Vulnerability Index; higher scores indicate more vulnerability

^e^Indicates dominant SARS-CoV-2 variant at time of infection

^f^Based on Charlson Comorbidity Index; count of co-existing chronic conditions based on ICD-10 codes

^g^Clinical severity during acute COVID-19 derived from hospitalization and oxygen support data

### Complete-case

In complete-case analyses, most variables were significantly associated with RID (Table 2). Smoking status was positively associated with post-COVID-19 RID with current and former smokers having a 10% (*p* = 0.15) and 30% (*p* < 0.001) higher odds of RID, respectively, compared with never smokers. Additionally, each additional comorbidity was associated with 18% lower odds of post-COVID RID (95% CI: 0.78, 0.87) and each additional COVID-19 vaccine dose increased odds of RID (OR: 1.10; 95% CI: 0.87, 1.40), although results were imprecise. Those with Dual Medicare/Medicaid had significantly higher odds of RID (OR: 1.58; 95% CI: 1.27, 1.98) compared to those with private insurance. Conversely, being uninsured was associated with a significantly lower odds of RID (OR: 0.52; 95% CI: 0.42, 0.63). Males had a non-significant lower odds of post-COVID RID compared to females, (OR: 0.94; 95% CI: 0.83, 1.05). Compared to those with the original COVID-19 variant, those with the Alpha (OR: 0.76, *p* = 0.003), Delta (OR: 0.90), and Omicron (OR: 0.89) variants all had significantly lower odds of post-COVID RID, though results for Delta and Omicron were imprecise. Severity of acute COVID-19 was also inversely associated with RID with 98% lower odds for those hospitalized and non-critical and hospitalized and critical, compared with only ambulatory care (all *p* < 0.001). Race/ethnicity was not statistically associated with RID.

**Table 2:** Complete-case analysis: association between demographic, clinical, social and COVID-related predictors and post-COVID respiratory infection diagnosis, odds ratios and 95% confidence intervals calculated from logistic regression estimates (*N* = 17,539)

Variable	OR (95% CI)	*p*-value^a^
Sex		
Female Male	Ref 0.94 (0.83, 1.05)	0.300
Race/Ethnicity		
White, non-Hispanic Black/African American, non-Hispanic Other^b^ Any race, Hispanic	Ref 0.88 (0.75, 1.03) 0.84 (0.67, 1.11) 0.93 (0.78, 1.11)	0.110 0.400 0.142
Insurance Status		
Private insurance Medicare Medicaid, CHIPS, or other non-military public Dual Medicare/Medicaid Other^c^ Uninsured	Ref 1.24 (0.99, 1.55) 1.16 (0.95, 1.41) 1.58 (1.27, 1.98) 0.70 (0.48, 1.01) 0.52 (0.42, 0.63)	0.065 0.140 < 0.001 0.072 < 0.001
Smoking Status^d^		
Never Former Current	Ref 1.10 (0.97, 1.26) 1.33 (1.13, 1.57)	0.150 < 0.001
Age	0.99 (0.98, 0.99)	0.029
Social Vulnerability Index^e^	0.97 (0.93, 1.00)	0.079
Body Mass Index	0.99 (0.98, 1.00)	0.005
COVID-19 Variant^f^		
Original Alpha Delta Omicron	Ref 0.76 (0.63, 0.91) 0.90 (0.74, 1.02) 0.89 (0.78, 1.02)	0.003 0.300 0.088
Total Comorbidities^g^	0.82 (0.78, 0.87)	< 0.001
Number of COVID-19 Vaccinations	1.10 (0.87, 1.40)	0.400
Severity Status due to COVID-19^h^		
Ambulatory Hospitalized, not critical Hospitalized, critical disease	Ref 0.02 (0.02, 0.02) 0.02 (0.02, 0.03)	< 0.001 < 0.001
Age * Number of COVID-19 Vaccinations	1.0 (1.00, 1.00)	0.800
Total Comorbidities * Severity (Ref: Ambulatory)		
Total Comorbidities * Hospitalized, not critical Total Comorbidities * Hospitalized, critical	1.70 (1.60, 1.81) 1.59 (1.45, 1.74)	< 0.001 < 0.001

^a^P-values smaller than 0.001 are denoted as < 0.001

^b^Other race category includes Asian, American Indian, Native Hawaiian, Other and Unknown race

^c^Other insurance category in Track PCC EHR data includes Temple-specific categories such as “Temple Athletic”, “Temple Med Student”, or “worker’s comp”

^d^Self-reported smoking behavior categorized as never smoker, former smoker, and current smoker

^e^Based on the CDC Social Vulnerability Index; higher scores indicate more vulnerability

^f^Indicates dominant SARS-CoV-2 variant at time of infection

^g^Based on Charlson Comorbidity Index; count of co-existing chronic conditions based on ICD-10 codes

^h^Clinical severity during acute COVID-19 derived from hospitalization and oxygen support data

### SuperMICE

Table 3 provides the SuperMICE MI-based estimates for our logistic regression model, all of which were statistically significantly associated with post-COVID-19 RID. Current (OR: 1.24) smokers had greater odds of post-COVID RID compared with never smokers (*p* < 0.001). Higher comorbidity count (OR: 0.88; 95% CI: 10.85, 0.92) was associated with a lower odds of RID. COVID-19 vaccinations were associated with a higher odds of RID (OR: 1.37; 95% CI: 1.11, 1.69). The Omicron variant was not associated post-COVID-19 RID, whereas Alpha and Delta were respectively associated with reduced odds (OR: 0.70 and 0.75, *p* < 0.001) compared with the original variant. Male sex was associated with lower odds of RID compared with females (OR: 0.89; 95% CI: 0.83, 0.97). All race/ethnicity groups had statistically significantly lower odds of RID compared with white, non-Hispanics. All types of public insurance, except Medicare (OR: 1.07, *p* = 0.457), (i.e., Medicaid/CHIPS/other non-military public insurance (19%), and Dual Medicare/Medicaid (42%)), had significantly increased odds of RID compared to private insurance. No insurance showed reduced odds of RID (*p* < 0.001). Increasing age (OR: 0.99), higher SVI (OR: 0.98), and higher BMI (OR: 0.98) were minimally associated with decreased odds of RID. Finally, hospitalized for COVID-19, both non-critical (OR: 0.04; 95% CI: 0.03, 0.04) and critical cases (OR: 0.05; 95% CI: 0.04, 0.06), had dramatically lower odds of post-COVID-19 RID compared with those solely with ambulatory care.

**Table 3:** SuperMice analysis: association between demographic, clinical, social and COVID-related predictors and post-COVID respiratory infection diagnosis, odds ratios and 95% confidence intervals calculated from multiple imputation logistic regression estimates

Variable	OR (95% CI)	*p*-value^a^	FMI^b^
Sex			
Female Male	Ref 0.89 (0.83, 0.97)	0.005	0.002
Race/Ethnicity			
White, non-Hispanic Black/African American, non-Hispanic Other^c^ Any race, Hispanic	Ref 0.89 (0.79, 0.99) 0.76 (0.64, 0.92) 0.85 (0.79, 0.99)	0.038 0.004 0.013	0.004 0.009 0.008
Insurance Status			
Private insurance Medicare Medicaid, CHIPS, or other non-military public Dual Medicare/Medicaid Other^d^ Uninsured	Ref 1.07 (0.89, 1.28) 1.19 (1.02, 1.38) 1.42 (1.19, 1.69) 0.81 (0.63, 1.05) 0.36 (0.31, 0.41)	0.457 0.023 0.001 0.105 < 0.001	0.001 0.001 0.001 0.001 0.002
Smoking Status^e^			
Never Former Current	Ref 1.05 (0.95, 1.17) 1.24 (1.12, 1.38)	0.301 < 0.001	0.002 0.001
Age	0.99 (0.99, 1.00)	0.670	0.001
Social Vulnerability Index^f^	0.98 (0.96, 1.00)	0.123	0.014
Body Mass Index	0.98 (0.98, 0.99)	< 0.001	0.001
COVID-19 Variant^g^			
Original Alpha Delta Omicron	Ref 0.70 (0.62, 0.80) 0.75 (0.66, 0.85) 1.01 (0.91, 1.69)	< 0.001 < 0.001 0.888	0.001 0.002 0.003
Total Comorbidities^h^	0.88 (0.85, 0.92)	< 0.001	0.000
Number of COVID-19 Vaccinations	1.37 (1.11, 1.69)	0.003	0.005
Severity Status due to COVID-19^i^			
Ambulatory Hospitalized, not critical Hospitalized, critical	Ref 0.04 (0.03, 0.04) 0.05 (0.04, 0.06)	< 0.001 < 0.001	0.002 0.007
Age * Number of COVID-19 Vaccinations	0.99 (0.99, 1.00)	0.045	0.009
Total Comorbidities * Severity (Ref: Ambulatory)			
Total Comorbidities * Hospitalized, not critical Total Comorbidities * Hospitalized, critical	1.51 (1.43, 1.58) 1.35 (1.27, 1.44)	< 0.001 < 0.001	0.008 0.009

^a^P-values smaller than 0.001 are denoted as < 0.001

^b^Fraction of missing information

^c^Other race category includes Asian, American Indian, Native Hawaiian, Other and Unknown race

^d^Other insurance category in Track PCC EHR data includes Temple-specific categories such as “Temple Athletic”, “Temple Med Student”, or “worker’s comp”

^e^Self-reported smoking behavior categorized as never smoker, former smoker, and current smoker

^f^Based on the CDC Social Vulnerability Index; higher scores indicate more vulnerability

^g^Indicates dominant SARS-CoV-2 variant at time of infection

^h^Based on Charlson Comorbidity Index; count of co-existing chronic conditions based on ICD-10 codes

^i^Clinical severity during acute COVID-19 derived from hospitalization and oxygen support data

### Interaction

In the SuperMICE analysis, a statistically significant interaction was observed between age and number of COVID-19 vaccine doses (*p* = 0.045), suggesting that the association between vaccine doses and PCC RID may vary across age groups. However, the interaction was not statistically significant in the complete-case analysis (*p* = 0.800).

Statistically significant interactions between total comorbidity burden and COVID-19 severity were observed in both the SuperMICE (*p* < 0.001) and complete-case analyses (*p* < 0.001). These findings indicate that the relationship between comorbidity burden and PCC RID differs according to the severity of the initial COVID-19 infection.

## Discussion

In this passive surveillance study of patients with prior COVID-19 infection in a diverse, urban health system, we found several patient-level and clinical factors were significantly associated with RID following COVID-19 infection. Results were generally consistent across complete-case and SuperMICE-based MI analyses. Smoking status, comorbidity burden, and vaccination history were associated with increased odds of post-COVID RID, whereas male sex, and hospitalization during acute COVID-19 were associated with reduced risk. Public vs. private insurance increased odds of post COVID-19 RID; whereas no insurance vs. private decreased risk of RID. In MI inference, we observed stronger associations in the same direction for all variables compared with the complete-case analysis except for COVID-19 variant Omicron. Specifically, those with Omicron had an increased odds of RID compared with the original variant, which was not observed in the complete-case analysis. Since the complete-case analysis is only valid under the “missing completely at random” (MCAR) assumption, it is likely to be misleading as this finding may be due to selection biases resulting from omitted cases pertaining to the variants. Race/ethnicity and BMI results differed between models but were directionally consistent and reached statistical significance among the SuperMICE models, likely due to recovered statistical information (e.g., gained statistical efficiency).

Our findings add to a growing body of evidence that COVID-19 survivors remain at heightened risk of subsequent respiratory morbidity. Prior studies have identified structural lung changes, immune dysregulation, and chronic inflammation as potential biological pathways [16, 33]. The associations we observed with smoking and multimorbidity align with existing evidence that both impair host defenses and increase susceptibility to respiratory pathogens [34–37]. Similarly, prior studies have observed elevated risks of lower respiratory tract infection and non-COVID respiratory illness following SARS-CoV-2 infection [10, 18]. Our results extend these findings by identifying population subgroups at greatest risk by leveraging a large, diverse EHR-based cohort.

Sociodemographic factors, including insurance status and race/ethnicity, can impact how patients interact with the healthcare system, introducing outcome ascertainment bias in EHR-cohorts [38]. Individuals with public insurance or those lacking coverage have structural barriers to ambulatory primary care, making them less likely to present to outpatient clinics for transient or mild post-COVID respiratory symptoms compared with insured populations. Similarly, racial and ethnic minoritized populations face health system disparities, including documented variations in clinical engagement and EHR documentation density. This differential healthcare-seeking behavior and health system engagement limits true incidence of subsequent respiratory infections [39]. These infections are likely under-coded among minoritized and publicly insured groups [40], attenuating the observed risk reflective of a selection bias and outcome misclassification. Finally, the effect of biological sex observed likely reflects a combination of true underlying sexual dimorphism and gender differences in outpatient healthcare utilization. Prior research indicates that females are significantly more likely to seek medical care and maintain primary care long-term [41–44]. In a passive surveillance framework, these increased clinical interactions elevate the probability that respiratory complaints are captured and diagnostic codes are recorded for females compared with males.

The inverse association between age and post-COVID RID although small is unexpected, given that advanced age is typically a risk factor for respiratory morbidity [45]. One explanation may be differential healthcare-seeking or diagnostic coding patterns across age groups, where younger or middle-aged adults in this cohort may interact differently with outpatient services for respiratory complaints. Alternatively, older adults may have received more consistent vaccination or preventive care, partially mitigating their risk. Unfortunately, this cannot be directly examined in the present study as vaccines are typically under-reported in EHR, and under-reporting is even more pronounced for COVID-19 [46–48]. In the present study, COVID-19 vaccination records were available for approximately 60% of patients. This limitation reflects known gaps in healthcare datasets, as missing vaccination records do not necessarily indicate a lack of vaccination, particularly given that many COVID-19 vaccinations occurred outside integrated health systems and may be under-documented. Although a positive association was observed between number of COVID-19 vaccine doses and odds of post-COVID respiratory infection, many individuals likely received doses outside of integrated systems and so this finding should be interpreted with caution [49]. Prior studies have shown that vaccination is protective against severe COVID-19 outcomes and may lower risks of certain PCCs [46, 50], suggesting that our observed positive association between vaccine doses and RID likely reflects confounding by healthcare utilization rather than a true biological effect. Patients who proactively seek out multiple doses within a health system may be more likely to frequently utilize healthcare services, thereby increasing the probability that subsequent respiratory infections are clinically captured and coded in EHR [51]. This is confounding by indication, which EHR are prone to when studying vaccine effectiveness, a limitation of using real-world EHR data to study vaccine-related outcomes [51].

The observed reduced risk among hospitalized COVID-19 patients also warrants consideration. Biologically, severe acute infection requiring hospitalization is typically tied to higher risks of sequelae [19, 52]. However, our inverse finding may reflect closer medical monitoring and prophylactic interventions during hospitalization, or alternatively, survivor bias or underreporting of subsequent respiratory infections among patients with more severe baseline disease who may have transitioned to long-term care facilities [53, 54], or experienced competing risks of mortality that removed them from our passive surveillance. [55].

Interaction analyses suggested that the relationship between the included correlates and PCC RID may differ across patient subgroups. The consistent interaction observed between total comorbidities and COVID-19 severity indicates that the impact of underlying health conditions on PCC RID risk may be amplified among individuals who experience more severe COVID-19 illness. This aligns with prior large cohort studies and meta-analyses of PCCs, which have identified both pre-existing comorbidities and markers of severe acute illness as key predictors of PCCs, including long-term respiratory and multi-organ morbidity [20, 56]. We also observed evidence of an interaction between age and vaccine doses in the SuperMICE analysis; however, this finding was not replicated in the complete-case analysis. Consequently, the potential modifying role of age on the relationship between vaccine doses and PCC RID should be interpreted cautiously and warrants further investigation. Although prior literature has reported reduced PCC among those vaccinated for COVID-19, heterogeneity has been observed in estimates, with variability across populations and study designs [57, 58]. An additional consideration is the presence of missing data for our vaccine doses variable, which may have influenced the stability of estimates. Although multiple imputation using SuperMICE was employed to reduce bias and improve efficiency under a missing-at-random assumption, differences between imputed and complete-case results suggest some sensitivity of interaction estimates to the handling of missing data. The non-significant age and vaccine dose interaction observed in the complete-case model may reflect reduced statistical power and potential selection bias introduced by complete-case restriction. Missingness in vaccination history may be related to healthcare utilization patterns or variability in clinical reporting which could not be evaluated in this study [59]. Together, these findings are consistent with prior literature and suggest that PCC RID risk hay be heterogeneous across populations supporting the need for future studies examining subgroup analyses.

This study has several notable strengths. First, leveraging the Track PCC surveillance project Temple Health data provided a large, racially and socioeconomically diverse sample. Additionally, the use of harmonized EHR and public health data enhanced statistical power and generalizability. An advanced imputation strategy (SuperMICE) was implemented that integrated machine learning to address missing data, improving the robustness of the findings compared to traditional complete-case analyses.

Several limitations include missingness due to the use of EHR data and ICD diagnoses. Missingness for RID and vaccination status likely reflects incomplete documentation rather than true representations of absence of exposure and disease [47, 48, 60]. Although SuperMICE mitigates some of these issues through sophisticated imputation techniques, residual bias is possible [61, 62]. Outcome misclassification is also a concern as we relied on ICD codes and medication data to identify respiratory infections; asymptomatic or untreated cases may not have been captured. In addition, the findings are restricted to individuals with at least one healthcare encounter, potentially underrepresenting those with limited access to care. Further, while there are limited opportunities for traditional confounding in the present analysis, important potential predictors were likely excluded due to the limited availability of individual-level data on diet, lifestyle, and other factors that could impact respiratory infection risk. Although correlates were selected based on prior literature, clinical relevance, and hypothesized relationships represented in our DAG, we cannot exclude the possibility of overadjustment or collider stratification bias. In particular, variables such as hospitalization status, disease severity, vaccination history, and healthcare utilization may be influenced by unmeasured factors related to baseline health status and healthcare access, which are not directly captured in our dataset. Consequently, the observed association should be interpreted cautiously as adjusted associations rather than causal effects.

Taken together, our results demonstrate that post-COVID respiratory infections are influenced by a combination of behavioral, clinical, and sociodemographic factors. Recognizing RID as a distinct post-COVID outcome has important implications for risk stratification, clinical follow-up, and health equity. Populations with smoking history, high comorbidity burden, and socioeconomic disadvantage may benefit from improved surveillance and preventive strategies. Integrating advanced analytic approaches such as SuperMICE into public health research can improve the reliability of EHR-based studies and inform tailored interventions aimed at mitigating long-term respiratory complications of COVID-19.

## Supplementary Information

Below is the link to the electronic supplementary material.

Supplementary Material 1

Supplementary Material 2

## Data Availability

The datasets generated and/or analyzed during the current study are not publicly available due to ongoing data collection but are available from the corresponding author on reasonable request.

## Abbreviations

Track PCC: Tracking the Burden, Distribution, Impact of Tracking Post-COVID-19 Conditions in Diverse Populations for Children, Adolescents, Adults passive surveillance cohort
COVID-19: SARS-CoV-2 infection
OR: Odds ratio
PCC: Post-COVID condition
EHR: Electronic health record
CDC: Centers for Disease Control and Prevention
ICD: International classification of diseases
PCR: Polymerase-chain reaction test
SVI: Social Vulnerability Index
DAG: Directed acyclic graph
RID: Respiratory infection diagnosis
BMI: Body mass index
CCI: Charlson Comorbidity Index
SD: Standard deviation
IQR: Interquartile range
SuperMICE: Supervised Multiple Imputation by Chained Equations
MICE: Multiple Imputation by Chained Equations
MCAR: Missing completely at random
