# Supplementary material for: Investigating respiratory infection as a post-COVID-19 condition using a large passive surveillance cohort from Track PCC
Source: BMC Infect Dis. 2026 Jul 16;26:1796. doi: 10.1186/s12879-026-13994-0 (PMC13660261; doi:10.1186/s12879-026-13994-0)
Supplement: Supplementary file 1 [file 12879_2026_13994_MOESM1_ESM.pdf]

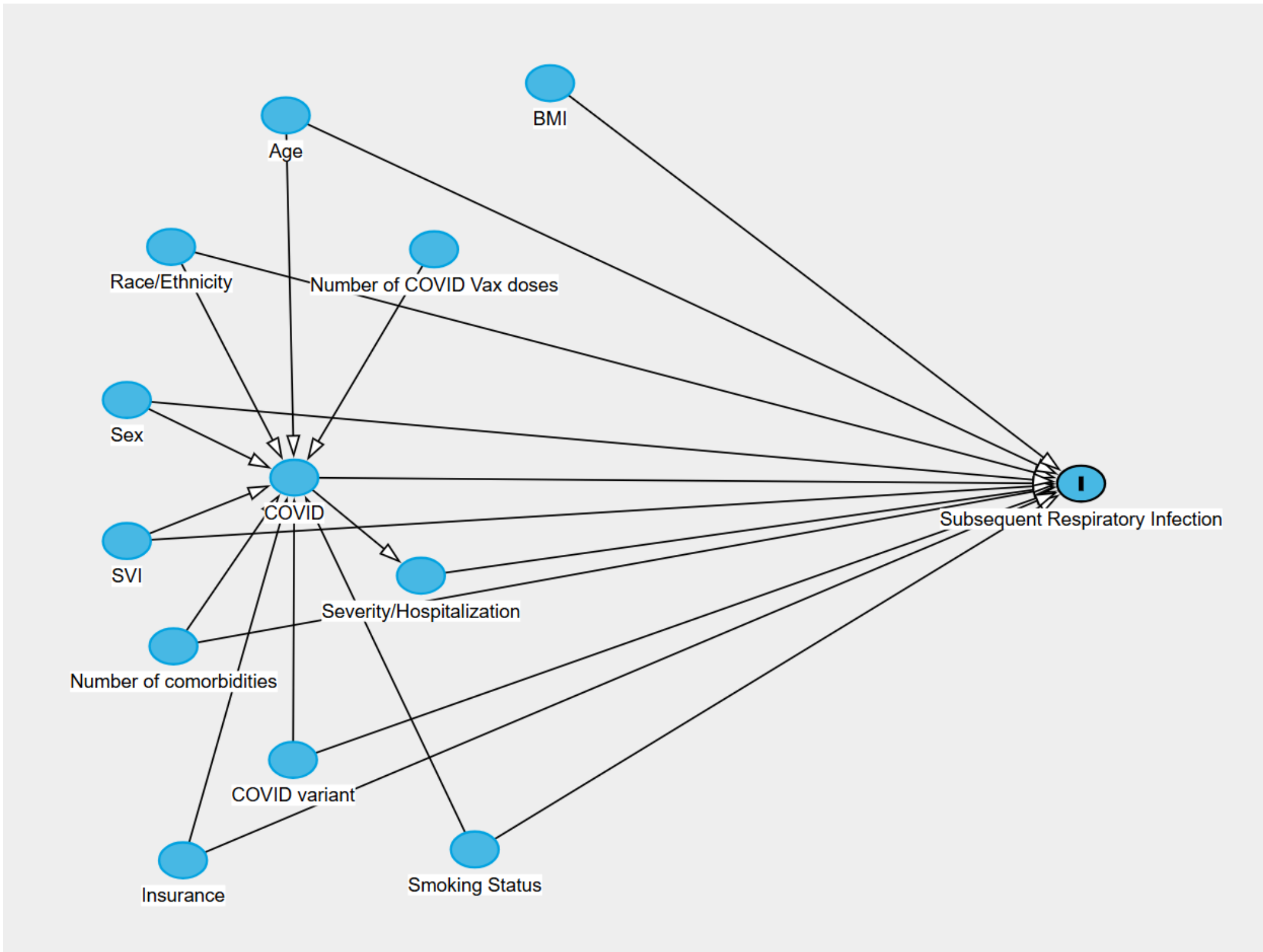

Supplemental Figure 1. Directed Acyclic Graph (DAG) illustrating assumed causal relationships and covariate selection

ALT TEXT: Directed acyclic graph depicting the assumed causal relationships between COVID and subsequent respiratory infection with hypothesized confounding and mediating variables.
