# Supplementary material for: Investigating respiratory infection as a post-COVID-19 condition using a large passive surveillance cohort from Track PCC
Source: BMC Infect Dis. 2026 Jul 16;26:1796. doi: 10.1186/s12879-026-13994-0 (PMC13660261; doi:10.1186/s12879-026-13994-0)
Supplement: Supplementary file 2 [file 12879_2026_13994_MOESM2_ESM.docx]

| **Supplementary Table 1. List of all ICD-10 codes included for indication of respiratory infection** | |
| --- | --- |
| **ICD-10 Code** | **Description** |
| J189 | Pneumonia, unspecified organism |
| J4540 | Moderate persistent asthma, uncomplicated |
| J45901 | Unspecified asthma with (acute) exacerbation |
| J4541 | Moderate persistent asthma with (acute) exacerbation |
| J45998 | Other asthma |
| J9809 | Other respiratory disorders |
| J4521 | Mild persistent asthma with (acute) exacerbation |
| J9601 | Acute respiratory failure with hypoxia |
| J1282 | Pneumonia due to coronavirus disease |
| J45909 | Unspecified asthma, uncomplicated |
| J9602 | Acute respiratory failure with hypercapnia |
| J80 | Acute respiratory distress syndrome |
| J811 | Acute pulmonary edema, unspecified |
| J90 | Pleural effusion, not elsewhere classified |
| J984 | Other disorders of lung |
| J209 | Acute bronchitis, unspecified |
| J40 | Bronchitis, not specified as acute or chronic |
| J1289 | Other viral pneumonia |
| J690 | Pneumonitis due to inhalation of food and vomit |
| J449 | Chronic obstructive pulmonary disease, unspecified |
| J15211 | Pneumonia due to Methicillin susceptible Staphylococcus aureus |
| J9611 | Chronic respiratory failure with hypoxia |
| J9690 | Respiratory failure, unspecified, unspecified |
| J9811 | Atelectasis |
| J4530 | Mild persistent asthma, uncomplicated |
| J4520 | Mild intermittent asthma, uncomplicated |
| J441 | Chronic obstructive pulmonary disease with (acute) exacerbation |
| J439 | Emphysema, unspecified |
| J154 | Pneumonia due to other streptococci |
| J810 | Congestive heart failure |
| J9600 | Acute respiratory failure, unspecified |
| J986 | Other diseases of mediastinum, not elsewhere classified |
| J22 | Unspecified acute lower respiratory infection |
| J849 | Pneumonia, unspecified |
| J121 | Respiratory syncytial virus pneumonia |
| J210 | Acute bronchiolitis due to respiratory syncytial virus |
| J1000 | Influenza with pneumonia, unspecified influenza virus |
| J9621 | Acute and chronic respiratory failure with hypoxia |
| J111 | Influenza due to unidentified influenza virus with other respiratory manifestations |
| J4489 | Other specified chronic obstructive pulmonary disease |
| J479 | Nasopharyngitis, unspecified |
| J129 | Viral pneumonia, unspecified |
| J440 | Chronic bronchitis, not specified as acute or chronic |
| J8410 | Pulmonary fibrosis, unspecified |
| J8489 | Other specified interstitial pulmonary diseases |
| J8283 | Eosinophilic pneumonia, unspecified |
| J9859 | Other diseases of bronchus, not elsewhere classified |
| J208 | Acute bronchitis due to other specified organisms |
| J9622 | Acute and chronic respiratory failure with hypercapnia |
| J9620 | Acute and chronic respiratory failure, unspecified |
| J9612 | Chronic respiratory failure with hypercapnia |
| J206 | Acute bronchitis due to rhinovirus |
| J438 | Other specified chronic obstructive pulmonary disease |
| J705 | Aspiration pneumonia |
| J988 | Other specified respiratory disorders |
| J9691 | Respiratory failure, unspecified with hypoxia |
| J8289 | Other specified pulmonary eosinophilia |
| J9801 | Bronchospasm, unspecified |
| J939 | Pneumothorax, unspecified |
| J4550 | Cough variant asthma |
| J4551 | Cough variant asthma with (acute) exacerbation |
| J84112 | Idiopathic pulmonary fibrosis |
| J101 | Influenza with other respiratory manifestations, unspecified influenza virus |
| J95811 | Postprocedural hematoma of respiratory system organ or structure following a respiratory system procedure |
| J159 | Unspecified bacterial pneumonia |
| J157 | Pneumonia due to Mycoplasma pneumoniae |
| J910 | Pleural plaque with presence of asbestos |
| J4531 | Mild persistent asthma with (acute) exacerbation |
| J181 | Lobar pneumonia, unspecified |
| J918 | Pleural effusion in conditions classified elsewhere |
| J4552 | Cough variant asthma with status asthmaticus |
